# Caregiver social support and child toilet training in rural Odisha, India: What types of support facilitate training and how?

**DOI:** 10.1111/aphw.12311

**Published:** 2021-10-19

**Authors:** Gloria D. Sclar, Hans‐Joachim Mosler

**Affiliations:** ^1^ Gangarosa Department of Environmental Health, Rollins School of Public Health Emory University Atlanta Georgia USA; ^2^ Department of Psychology University of Zürich Zürich Switzerland; ^3^ RanasMosler Zürich Switzerland

**Keywords:** childcare, India, perceived stress, self‐efficacy, social support, toilet training

## Abstract

Studies show positive impacts of social support on childcare practices, but there is limited research on child toilet training. Social support with toilet training may be especially important for rural Indian caregivers as this is a new childcare practice for many and mothers face an already demanding workload. The aim of this study was to examine the role of social support in toilet training using mediation and conditional process analyses. We surveyed 570 caregivers of children <5 years old living in rural Odisha, India. We found certain types of support aid toilet training through three mechanisms: directly, by improving self‐efficacy, and by buffering against stress. Informational and instrumental support had a positive direct effect on toilet training while emotional support had no effect. Instrumental support also aided toilet training indirectly through bolstering a caregiver's perceived self‐efficacy. These effects of instrumental support were not moderated by the caregiver's support network size. Additionally, we found perceived stress had a negative indirect effect on caregivers' toilet training efforts through diminishing self‐efficacy, but this effect was buffered (i.e. moderated) by social support. These findings offer useful programmatic insights and expand the evidence‐base on how social support functions to another childcare practice and cultural context.

## INTRODUCTION

Toddlerhood is a period of dynamic development that fosters an exhaustive exploratory nature well‐known to every caregiver. At this stage children begin to walk, squat, master fine motor skills, rapidly acquire language, understand norms and rules, and experience self‐conscious emotions like pride and shame (Packer, 2017). Toilet use is a momentous milestone of this development stage (Baird et al., 2019). But for many parents, toilet training is a stressful process; there are a variety of different training methods to choose from, with little evidence of a best approach, and complications can arise for the child such as stool toileting refusal, hiding to defecate, and nighttime bedwetting (Baird et al., 2019; Kiddoo, 2012; Vermandel et al., 2008). Programs and strategies that strengthen a caregiver's social support during this time may help the toilet training process be less stressful and more successful. Research to date has examined the role of social support in a variety of childcare practices but there is little focus on toilet training. Social support with toilet training may be particularly important for rural Indian mothers because toilet use is a relatively new practice, as the country attains greater sanitation access, and Indian women face a demanding workload making it potentially stressful to incorporate additional childcare tasks. There is a need for empirical evidence, especially from different cultural contexts such as India, to inform the design of caregiver support programs on this child development milestone.

Social support can be defined as the social resources our relationships provide to us, or we perceive are available to us, in times of need (Cohen et al., 2000). Social support can take many different forms including emotional acts of support (e.g., providing comfort, a listening ear, empathy, and encouragement), informational support (e.g., giving advice, feedback, and guidance), and instrumental support (e.g., tangible helpful acts and financial assistance) (Wills & Shinar, 2000). Studies show a wide‐range of positive and protective impacts of social support on parenting, including improvements in breastfeeding (Fox et al., 2015; Schmied et al., 2011), lower stress among parents of children with developmental disorders (Shepherd et al., 2020; Zaidman‐Zait et al., 2017), and better mental health and confidence for first‐time mothers (Chavis, 2016; Leahy‐Warren et al., 2012; Lee et al., 2020). For example, qualitative studies that explored breastfeeding challenges among mothers in the United Kingdom and United States found acts of emotional and informational support were critical for successful and sustained breastfeeding (Fox et al., 2015; Schmied et al., 2011). Like breastfeeding, toilet training requires commitment and daily routine, suggesting parents would benefit from social support with this childcare practice as well.

Social support is thought to influence behavior through two related mechanisms: it bolsters self‐efficacy and protects against stressors. In social cognitive theory, Albert Bandura theorized that a person needs to have confidence in their ability to perform a behavior, termed *self‐efficacy*, before actually doing the behavior. When individuals lack the level of self‐efficacy needed, social support techniques can be employed to help bolster the individual's perception of their ability (Bandura, 1986). This type of self‐efficacy has been called “action self‐efficacy.” Other types of self‐efficacy that social support may strengthen include “maintenance self‐efficacy” (confidence in one's ability to overcome barriers that arise) and “recovery self‐efficacy” (confidence in one's ability to continue with a behavior despite setbacks or experiences of failure) (Schwarzer, 2008). Empirical studies show these latter two forms of self‐efficacy are important for health behaviors that need to be sustained over time (Luszczynska & Schwarzer, 2003; Scholz et al., 2005). Toilet training can be viewed in this light; it is a dyad behavioral process that the child and caregiver must stay committed to until the child learns to independently use the toilet.

Social support can also function as a protective shield or “buffer” against stressors, known as the buffering hypothesis (Cohen & Wills, 1985). Social support acts as a buffer by strengthening a person's ability to cope with stress and appraise situations as less stressful (Cohen & Wills, 1985). In this way, social support not only helps an individual believe they can do a behavior but believe they can continue despite external stressors or take on more stressful behaviors, linking back to maintenance, and recovery self‐efficacy. The stress‐support matching hypothesis goes one step further to delineate that not all forms of social support will have a positive effect on a given stressor: the stressor must be matched with the specific type of support needed (Cohen & McKay, 1984; Cultrona & Russell, 1990). In fact, when an undesired form of support is given, however well‐intentioned, this can lead to negative effects on the receiver.

The relationships in a person's life, the potential providers of support, are another important aspect to consider. How integrated a person is in society and the characteristics of their social network influence how effective provided support will be in reducing stress and improving self‐efficacy (Brisette et al., 2000). For example, a large support network may allow for more opportunities of support while a diverse network may offer a greater variety of support. As one study in Andhra Pradesh showed, children had better nutritional outcomes when their mothers had larger and more literate social networks, suggesting such networks may be well‐suited to provide informational support on nutrition (Moestue et al., 2007).

In understanding the ways social support functions, it becomes clear how this social resource lends itself to parenting and childcare. Parents are constantly having to take on new responsibilities, master different skills, and navigate issues that arise as their child grows—all of which require confidence and an ability to manage stress. Many studies document the stressors of parenthood and how a lack of parental support can compound this stress while access to a supportive network can lead to better parenting practices and child development (Lorenz et al., 2020; McConnell et al., 2011; Parkes et al., 2015; Taylor et al., 2015). But there is surprisingly little research on how social support might aid the toilet training process.

Research on toilet training to date has mostly been descriptive, focusing on the age of initiation and different methods, with only a few studies on the caregiver experience. Two well‐known methods include the Brazelton child‐oriented approach, a gradual and positive promotion of toilet use when the child shows signs of readiness, and the Azrin and Foxx parent‐oriented approach, an intensive training that uses operant conditioning (Brazelton, 1962; Foxx & Azrin, 1973). The Indian Academy of Pediatrics, as well as others, promote aspects of the child‐oriented approach with the recommendation to start around 2‐years‐old (AAP, 1999; CPS, 2000; IAP, 2020). A qualitative study explored how Swedish parents navigated toilet training with a child‐oriented approach (Jansson et al., 2008). Parents described how “having time” was a major factor in their decision to start training, that the process required establishing “new daily routines” with their child, and feeling social pressure from an “unspoken rule” that their child should be fully trained by a certain age (Jansson et al., 2008). Another qualitative study among Belgian parents and childcare workers identified similar themes: Toilet training was viewed as time‐consuming and parents felt anxious about training their child in time for primary school (Van Aggelpoel et al., 2019). Parents also described the different forms of support they received; grandparents and teachers helped with the training process (i.e., instrumental support) and doctors were asked for trusted guidance (i.e. informational support).

Rural Indian caregivers in particular may need support with toilet training because for many, this is a new childcare task not practiced by previous generations. Over the past several decades India has achieved greater access to sanitation, with the proportion of the Indian population using a toilet increasing from 16% to 60% between 2000 and 2017 (JMP, 2019). As adults adopt toilet use, children who are likely developmentally ready to toilet train continue to defecate in the open. According to the 2015–2017 National Family Health Survey, only 37% of Indian children between 4 and 5‐years‐old used the toilet (NFHS, 2017). Toilet training as soon as the child is ready is especially important in rural India where young children often defecate around the household compound and caregivers unsafely dispose of the feces outside rather than in a toilet (Majorin et al., 2014; NFHS, 2017). This unsafe disposal of child feces has been linked to negative impacts on child growth and greater risk for diarrheal disease (Bauza & Guest, 2017; Bawankule et al., 2017). In areas with adequate sanitation and water access, young children are starting to learn to use the toilet. One study among rural villages in Odisha, India with high toilet and water coverage found 57% of 3‐ to 4‐year‐olds and 82% of 4‐ to 5‐year‐olds used the toilet (Bauza et al., 2019). As families start to toilet train their children for the first time, mothers and caregivers may need supportive guidance on when and how to navigate the toilet training process.

Social support with toilet training may also be important for Indian mothers due to their already demanding workload. A recent time‐use study by the Government of India found rural Indian women spend on average 132 min per day on unpaid caregiving labor and 301 min on unpaid domestic work—two to three times higher than their male counterparts (NSO, 2019). In addition, women continue to participate more and more in agricultural labor, facing severe time‐constraints between farming and household demands during harvest seasons (Pattnaik & Lahiri‐Dutt, 2021; Vemireddy & Pingali, 2021). Qualitative examinations reveal how this overwhelming workload with childcare, cooking, cleaning, and farming tasks even leads to maternal and child malnutrition (Chaturvedi et al., 2016; Chorghade et al., 2006). Indian mothers might find it stressful to incorporate toilet training into their daily workload and, as a result, desire instrumental support from others.

The aim of this study is to examine the role of social support in toilet training among rural Indian caregivers with household toilet and water access, in order to (1) help inform programming and (2) expand the evidence base on how social support functions to a new childcare practice and context.

**FIGURE 1:**
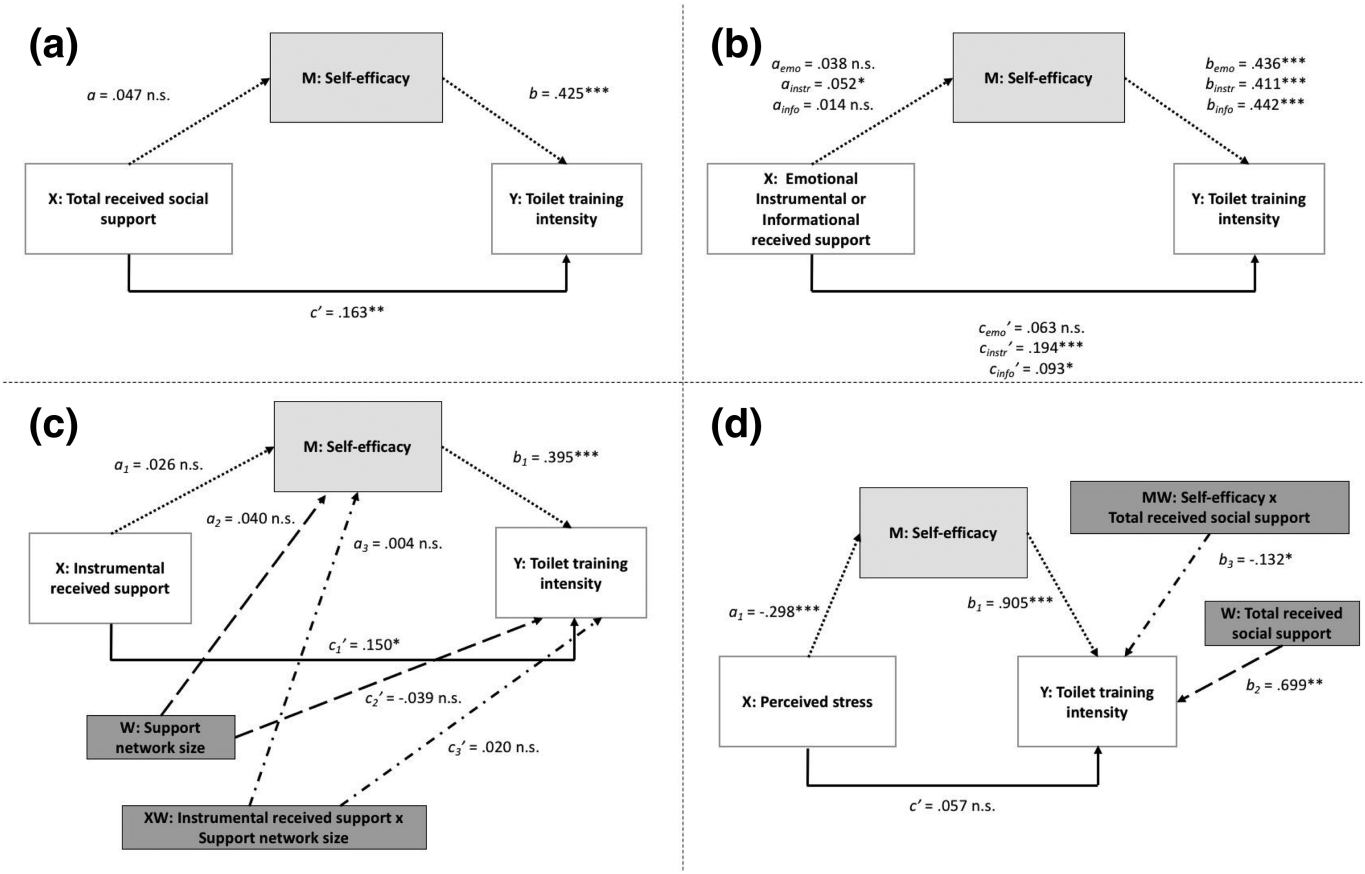
Statistical diagrams of simple mediation and moderated mediation analyses: (a) simple mediation analysis of total received social support; (b) simple mediation analysis for each specific type of social support; (c) moderated mediation with support network size as moderator; (d) moderated mediation with total received social support as moderator. *p < .05; **p < .01; ***p < .001

We examine the following hypotheses:

Hypothesis 1 Social support will be positively associated with toilet training intensity through bolstering the caregiver's perceived self‐efficacy; that is, self‐efficacy will mediate the effect between social support and toilet training (see Figure 1a for a visual of the hypothesis).

Hypothesis 2 The effect of social support on caregiver's toilet training intensity will be specific to certain types of support (Figure 1b). We hypothesize Indian caregivers will desire informational support on how to do toilet training, as it is a childcare practice not done by past generations, and instrumental support due to their already demanding workload.

Hypothesis 3 The mediated relationship between social support and toilet training intensity will be stronger for caregivers who have many people in their life providing social support compared to caregivers who have few people; that is, the size of the caregiver's social support network will moderate the mediated effect (Figure 1c).

Hypothesis 4 General life stress will be negatively associated with toilet training intensity through diminishing the caregiver's perceived self‐efficacy. This mediated relationship will be moderated, or buffered, by social support: The effect of stress on toilet training intensity will be weaker for caregivers with high levels of social support compared to caregivers with low levels of social support (Figure 1d).

## METHODS

### Study setting

This study is part of a larger cluster randomized trial evaluating a behavior change program that aims to improve safe child feces disposal and child toilet training among households in rural Odisha, India. The trial engages caregivers of children <5 years old who reside in Ganjam and Gajapati districts, which differ in both their geography and demography. Ganjam includes a mix of hills, valleys, tableland, and coastal plains, larger villages with closer access to markets and towns, and is home to a mostly Hindu population that is Other Backward Caste or General caste. In contrast, Gajapati is a more remote and hilly region with smaller villages and a primarily Scheduled Tribe population, many of whom are Christian. The trial enrolled 74 villages, 49 in Ganjam and 25 in Gajapati, that had previously participated in a community‐based water and sanitation program. As such, households have a twin‐pit pour flush latrine, bathing room, and piped water supply. This helped to ensure caregivers already had an enabling physical environment to perform safe child feces disposal and child toilet training. The data used in this analysis comes from the baseline trial survey administered prior to program implementation.

### Participants

In each trial village, all households with a child <5 years old and having a toilet were eligible to participate in the survey. The respondent was the primary caregiver of the child but if the primary caregiver was not available then a secondary caregiver was surveyed. Most respondents were the mother of the child but some fathers and grandmothers were also surveyed; as a result, we use the broader term “caregiver.”

### Survey and measures

The survey was administered in the local language Odia and consisted of six key sections: caregiver's perceived stress, current child feces management practices, psychosocial factors related to toilet training (i.e., risk perception of child feces and social norms around toilet training), received social support with toilet training, respondent demographics, and household water and sanitation facilities. Several survey items in the psychosocial factors and social support sections were constructed based on qualitative formative research involving in‐depth interviews and focus group discussions with mothers and secondary caregivers. We also conducted cognitive interviews with 13 mothers on these two sections, along with the perceived stress section, to check comprehension of survey items, contextual relevance, and whether or not items tapped the intended concept. The full survey was then pilot tested prior to data collection (see Table S1 for the survey items used in this analysis).

### Toilet training intensity

We used two items to measure how much a caregiver was focused on her child's toilet training, termed *toilet training intensity*. Caregivers were first asked “Are you currently teaching your child how to use the latrine for defecation?” with different clarifying response options (yes, no—my child is not old enough, no—my child already knows how to use the latrine, no—though my child is old enough). If the caregiver said yes, then a follow‐up was asked, “During the last week, when your child needed to defecate, how often did you take your child to the latrine and teach them how to use it?” with a five‐point Likert response option (1 = *(almost) never (0%)*, 5 = *(almost) always (100%)*). The first question assesses whether the caregiver herself perceives she has started toilet training with her child. The second question then measures the caregiver's toilet training efforts by determining how consistently she undertakes the training process. Toilet training intensity was thus measured as a score between 1 and 5 based on the answer to the second question, with a higher score indicating greater training intensity. Those caregivers who reported in the first question that they were not yet teaching their child how to use the toilet, though their child was old enough, were assigned a 1 for *(almost) never*.

### Perceived self‐efficacy

We used four items to capture a holistic measure of caregivers' perceived self‐efficacy around toilet training. The items cover three different forms of self‐efficacy: action self‐efficacy (“How confident are you in your ability to successfully teach your child how to use a latrine?”), maintenance self‐efficacy (“If your child needs to defecate in the nighttime and needs your help, how confident are you that you would go and help your child use the latrine?”; “How confident are you in your ability to teach your child how to use the latrine when there is a water shortage?”) and recovery self‐efficacy (“How confident are you in your ability to continue teaching your child how to use a latrine when your child refuses to use the latrine – for example, child cries or won't enter the latrine?”). All items had the same five‐point Likert response option (1 = *not confident*, 5 = *very confident*). The examples used in the maintenance and recovery self‐efficacy items were common barriers and setbacks that caregivers described during the qualitative formative research. Following confirmatory factor analysis (CFA) (see section below), a perceived self‐efficacy score from 1 to 5 was constructed with a higher score indicating greater self‐efficacy.

### Received social support

We used 13 items to measure how much support caregivers receive with toilet training. Each item addressed a specific type of social support: six items for emotional support, four items for instrumental support, and three items for informational support. Caregivers were prompted at the beginning to “Think about the different people in your life such as family members, neighbors and friends. What help did you receive in the last week with teaching your child to defecate in the latrine?” All items used the same agree/disagree six‐point Likert response option. During pilot testing some caregivers found the six‐point Likert difficult, so a visual aid was created to help caregivers select their answer. To our knowledge, there is no existing metric that specifically measures social support for child toilet training and particularly for the rural Indian context. As such, we developed our metric by adapting items from five validated social support scales (Barrera et al., 1981; Schwarzer & Schulz, 2013; Vaux et al., 1987; Zimet et al., 1988). We took the following approach: compiled all survey items across the five metrics, reviewed the items and broadly categorized into type of support measured, identified the distinct forms or acts of each support type commonly measured (e.g. emotional supportive acts included listening, comforting, empathizing, encouraging, and showcasing closeness and acceptance), selected acts of support most relevant to child toilet training, and adapted item wording accordingly. In addition, the qualitative formative findings were used to identify real and common examples of instrumental supportive acts (e.g., family member takes care of household cooking or cleaning tasks so the caregiver can spend time on toilet training). Following CFA (see section below), a total received social support score and support‐specific scores from 1 to 6 were constructed with a higher score indicating greater support.

### Support network size

After answering the social support survey items, caregivers were asked “For the statements that you agreed to, who were the people that provided you this support?” The social support network size then became the total number of unique individuals identified by the caregiver, including different family members, neighbors, and other sources of support.

### Perceived stress

We used the Perceived Stress Scale (PSS) by Cohen et al. (1983) to measure how much stress caregivers perceive they have in their life. The PSS was designed to be a globally applicable scale and has been deployed in a variety of settings and populations (Lee, 2012). We used the 10‐item scale (PSS‐10) rather than the original 14‐item scale (PSS‐14) because studies show it is more reliable and it helped reduce survey length (Lee, 2012; Leung et al., 2010). The PSS‐10 consists of six negative items and four positive items, each asking how often the respondent felt or thought a certain way in the last month, with a five‐point Likert response option (1 = *never*, 5 = *very often*). The items describe general situations for the respondent to appraise, which examine how uncontrollable, unpredictable, and overloaded the respondent perceives their life to be. The scale was translated into Odia by the research team and then tested using cognitive interviews to ensure each item retained its original meaning. The perceived stress score was calculated by first reverse scoring the four positive items and then averaging the 10 items to get an overall score between 1 and 5, with a higher score indicating greater stress.

### Data collection

A team of enumerators fluent in Odia, 10 women and one man, administered the surveys using the application Open Data Kit (ODK) Collect on an encrypted Android phone. The enumerator team underwent a five‐day training followed by pilot‐testing in three non‐study villages. The training included an orientation to key survey topics, review of all survey questions and response options, discussion of enumerator role and ethics, and ODK practice. During training enumerators provided critical feedback on the construction and translation of survey questions to improve respondent comprehension.

Data collection took place between December 2019 and February 2020. Enumerators went door‐to‐door to check household eligibility. Once eligibility was confirmed, the enumerator asked to speak to the primary caregiver of the child <5 years old and upon their consent, administered the survey. The survey typically lasted between 30 and 60 min. The field supervisor observed a subset of surveys throughout data collection to ensure quality.

### Confirmatory factor analysis: Social support and self‐efficacy scores

CFA was conducted for the received social support and self‐efficacy scores to confirm the survey items related to the intended construct and determine the score calculation accordingly. We chose CFA rather than exploratory factor analysis (EFA) because these psychological constructs have been heavily theorized, with the relationship between the items and given construct well‐understood (Knekta et al., 2019). As such, the factor models were prespecified (Figures S1 and S2). For received social support, a three‐factor model with 13 items was tested; each type of social support (i.e., emotional, informational, and instrumental) acted as a factor with all survey items intended to measure that given type of social support linking to the factor. For self‐efficacy, a one‐factor model with four items was tested with all items linking to the “self‐efficacy” factor. In both models, each factor had at least three items as recommended (Bandalos & Finney, 2019). No item had more than 2.3% missing data. All items had a skewness value less than |2.0| and kurtosis value less than |4.0|, suggesting normality. With the data assumed to be normal and continuous, maximum likelihood was used as the estimation method (Bandalos & Finney, 2019). Standard goodness‐of‐fit indices were employed to assess absolute and incremental model fit (Boateng et al., 2018; Schumacker & Lomax, 2004). The CFA analysis was performed in STATA v16.

The three‐factor model for social support with 13 items did not have adequate model fit (*χ* ^2^:df ratio = 5.957; RMSEA = 0.094 [0.085–0.103]; CFI = 0.892; TLI = 0.864). All standardized factor loadings were significant and between 0.433 and 0.870. It is debated at what factor loading an item should be dropped; some methodologists suggest factor loadings <0.32 are non‐salient (≤10% explained variance) (Tabachnick & Fidell, 2007), while others suggest factor loadings <0.71 are non‐salient (<50% explained variance) (Bandalos & Finney, 2019). To refine the model and improve fit, we considered removing only those items with comparatively lower factor loadings of <0.50 (<25% explained variance) and lacking a strong theoretical basis for inclusion. Three items were thus removed: two items related to emotional support (factor loadings 0.498 and 0.433) and one item related to instrumental support (factor loading 0.457) (Table S3). This refined three‐factor model with 10 items achieved adequate model fit (*χ* ^2^:df ratio = 3.974; RMSEA = 0.073 [0.060–0.086]; CFI = 0.959; TLI = 0.942) and had good factor loadings (between 0.541 and 0.892) (Table S3). Accordingly, the total received social support score was calculated by averaging the 10 items in the refined model; support‐specific sub‐scores were calculated for emotional, instrumental and informational support in the same manner using their relevant items from the refined model.

The one‐factor model for self‐efficacy with four items had adequate model fit (*χ* ^2^:df ratio = 3.858; RMSEA = 0.071 [0.023–0.126]; CFI = 0.989; TLI = 0.966). All standardized factor loadings were significant and between 0.583 and 0.721 (Table S4). The self‐efficacy score was calculated by averaging the four items.

### Mediation and moderation analyses

The study hypotheses propose both mediated and moderated effects. Hypotheses 1 to 3 were tested sequentially, building on findings from the previous and with social support as the independent variable and caregiver toilet training intensity as the outcome variable. Hypothesis 4 was tested with perceived stress as the independent variable. We first examined intercorrelations using Pearson's *r* to gather an initial understanding of the relationship between the variables. We then used the statistical application PROCESS in SPSS v26 by Preacher and Hayes (2004) to test our hypotheses. For tests of mediation, we used bootstrapped confidence intervals to determine significance of the indirect effect *ab*. This approach was chosen over the Sobel test as it does not rely upon a tenuous assumption of normality for the indirect effect distribution (Edwards & Lambert, 2007). In Hypotheses 3 and 4 we tested whether or not the strength of the mediated effect depends upon the value of a moderating variable; this is known as moderated mediation or a conditional indirect effect (Preacher et al., 2007). In both hypotheses the relationship between the indirect effect and moderator is linear. As such, we used the index of moderated mediation by Preacher et al. (Preacher et al., 2007), with bootstrapped confidence intervals, to first test for moderation. If there was evidence of moderation, we then probed the interaction using the Johnson‐Neyman Technique to identify value ranges of the moderator where the moderated mediation was significant, known as “regions of significance” (Johnson & Fay, 1950).

## RESULTS

### Caregiver demographics and child toilet training

A total of 1033 caregivers of children <5 years old were surveyed but 461 caregivers were dropped from the analysis: 24 ended the survey early, 41 did not consent, 346 reported their child was not yet old enough to use the toilet (most had children between 0 and <2 years old), 46 reported their child was already using the toilet on their own (almost all had children ≥2 years old), and six refused to answer the toilet training question. This left 570 caregivers in the analysis.

Among these 570 caregivers, most were the mother of the child (86.7%), had only one child <5 years old (81.9%), were currently married (97.3%), between the ages of 18 to 30 years old (73.9%), and had a ration card for food assistance (84.5%) (Table S2). Caregivers varied in their level of education: 23.1% never attended school, 34.8% had a primary education (between grades 1–8), and 37.1% had a secondary education (between grades 9–12). About half of caregivers were unemployed while 39.1% were self‐employed, such as with agricultural works, and 11.1% were employed outside the home. With regard to household characteristics, caregivers belonged to households that were either Hindu (79.4%) or Christian (20.1%) and primarily Other Backward Caste (34.6%), Scheduled Tribe (25.8%) or General caste (17.7%). Since caregivers lived in villages that had previously participated in a community‐based water and sanitation program, most households had a bathing room (97.1%) and functional piped water supply (88.4%), and very few shared their pour‐flush latrine with other households (4.5%).

The children of the caregivers were 50.9% female and typically between 2 and <5 years old (89.8%). The average age at which caregivers first started to teach their child how to use the toilet was 2‐years‐old. When asked how often in the last week they took their child to the toilet and taught them how to use it, 47.4% of caregivers reported (almost) always, 7.7% often, 20.7% sometimes, 4.2% seldom, and 20.0% (almost) never.

### Intercorrelations of study variables

The Pearson correlations give an initial impression that the hypothesized relationships between the variables, for both mediated and moderated effects, may be present (Table 1). Toilet training intensity was positively related to self‐efficacy (*r* = .23, *p* < .01) and received social support, for both total social support (*r* = .16, *p* < .01) and each of the three specific types of support: emotional (*r* = .09, *p* < .05), instrumental (*r* = .22, *p* < .01), and informational (*r* = .11, *p* < .01). However, only instrumental support was positively related to self‐efficacy (*r* = .09, *p* < .05). Perceived stress was negatively related with self‐efficacy (*r* = −.19, *p* < .01), total received social support (*r* = −.11, *p* < .05), and emotional support (*r* = −.13, *p* < .01), but not significantly related with toilet training intensity or the other types of support. In contrast, support network size was positively related to all relevant study variables: toilet training intensity (*r* = .14, *p* < .01), self‐efficacy (*r* = .11, *p* < .05), total received social support (*r* = .36, *p* < .01), emotional support (*r* = .24, *p* < .01), instrumental support (*r* = .37, *p* < .01), and informational support (*r* = .30, *p* < .01).

**TABLE 1:** Descriptive statistics and Pearson correlations for study variables

Variable	n	Range	Mean	SD	1	2	3	4	5	6	7
1. Toilet training intensity^+^	570	1–5	3.58	1.57							
2. Total received social support	519	1–6	3.84	1.36	**.16** **						
3. Emotional support	528	1–6	3.47	1.73	**.09** *	**.84** **					
4. Instrumental support	544	1–6	4.62	1.55	**.22** **	**.71** **	**.35** **				
5. Informational support	531	1–6	3.54	1.77	**.11** **	**.84** **	**.54** **	**.48** **			
6. Self‐efficacy	547	1–5	3.99	0.86	**.23** **	.07	.07	**.09** *	.02		
7. Perceived stress	554	1–5	2.92	0.52	−.03	**−.11** *	**−.13** **	−.04	−.06	**−.19** **	
8. Support network size	565	0–7	2.02	1.22	**.14** **	**.36** **	**.24** **	**.37** **	**.30** **	**.11** *	−.07

* *p* < .05.

** *p* < .01.

### Hypothesis 1

In Hypothesis 1, we applied Bandura's social cognitive theory to predict that received social support is positively associated with toilet training intensity, in part through bolstering the caregiver's sense of self‐efficacy in her ability to perform toilet training. The statistical diagram and corresponding unstandardized regression coefficients of the hypothesized simple mediation are presented in Figure 1a and the total, direct, and indirect effects are presented in Table 2.

**TABLE 2:** Summary of simple mediation analyses with different forms of social support as the predictor and toilet training intensity as the outcome variable

Predictor	Total effect			Direct effect			Indirect effect		
	B	SE	p	B	SE	p	B	SE	Boot 95% CI
Total support	**.183** ***	.050	.000	**.163** **	.049	.001	.020	.013	(−.003, .049)
Emotional support	**.079** *	.040	.046	.063	.039	.104	.017	.010	(−.002, .038)
Instrumental support	**.215** ***	.042	.000	**.194** ***	.042	.000	**.021**	.011	(.001, .045)
Informational support	**.099** *	.038	.010	**.093** *	.037	.013	.006	.010	(−.012, .026)

*Note*: *n* = 516/ 524/540/528; Bootstrap sample size = 10,000.

* *p* < .05.

** *p* < .01.

*** *p* < .001.

Total received social support was not significantly associated with self‐efficacy, but self‐efficacy was positively associated with toilet training intensity (*b* = .425, *p* < .001). As predicted, total received social support had a positive direct effect on toilet training intensity (*c′* = .163, *p* < .01). However, it did not have an indirect effect on toilet training intensity through self‐efficacy. The bootstrapped 95% confidence interval around the indirect effect contained zero (*B* = .020, Boot 95% CI: −.003, .049).

### Hypothesis 2

In Hypothesis 2, we applied the stress‐support matching hypothesis to theorize that not all forms of social support would be associated with toilet training intensity. Instead, we hypothesized caregivers would specifically desire instrumental and informational social support to help them with toilet training. The statistical diagram and corresponding unstandardized regression coefficients of the hypothesized simple mediation for each specific type of social support are presented in Figure 1b and the total, direct, and indirect effects are presented in Table 2.

Instrumental support was the only type of social support positively associated with self‐efficacy (*a*_*instr*_ = .052, *p* < .05), while self‐efficacy was again positively associated with toilet training (*b* = .411 to .442, *p* < .001). Instrumental and informational support had a positive direct effect on toilet training intensity (*c'*_*instr*_ = .194, *p* < .001; *c'*_*info*_ = .093, *p* < .05), but emotional support had no significant direct effect. Instrumental support also had a positive indirect effect on toilet training intensity through self‐efficacy with a bootstrapped 95% confidence interval around the indirect effect that did not contain zero (*B* = .021, Boot 95% CI: .001, .045).

Taking the results of Hypotheses 1 and 2 together, we find that received social support has a positive direct effect on caregiver's toilet training intensity but this effect is indeed support‐specific, operating primarily through instrumental support and some through informational support. Instrumental support also indirectly affects a caregiver's toilet training intensity through bolstering her sense of self‐efficacy.

### Hypothesis 3

To build on these findings, we next examined if the size of a caregiver's social support network *moderates* the uncovered direct and indirect effects of instrumental support on toilet training—that is, if the effects are *conditioned* upon the size of the caregiver's support network. We hypothesized that both the direct and indirect effects would be stronger for caregivers who have many people in their life providing instrumental support compared to caregivers who have few people in their life providing this support. Caregivers' support networks ranged in size from zero to seven people: 33.8% described only one person who provided them with this support, 50.6% said two to three people, 11.3% said four to seven people, and 3.7% said no one. The most common sources of support were family members such as grandmothers (58.1%), husbands (55.7%), grandfathers (21.3%), and aunts (16.9%). Few caregivers mentioned their neighbors (8.6%), the local childcare center worker (2.8%), or community health worker (2.0%). The statistical diagram and corresponding unstandardized regression coefficients of the hypothesized conditional effects are presented in Figure 1c and the interaction terms are presented in Table 3.

**TABLE 3:** Summary of moderated mediation analyses with toilet training intensity as the outcome variable

Hypothesis 3: Support network size as moderator			
Interaction terms	B	SE	p
Conditional direct effect:			
*Instrumental support x support network size*	.004	.022	.867
Conditional indirect effect: *Instrumental support x support network size*	.020	.038	.596
	**Index of moderated mediation**	**Boot *SE***	**Boot 95% CI**
*Support network size*	.001	.009	(−.017, .020)

Hypothesis 4: Total received social support as moderator			
Interaction term	*B*	*SE*	*p*
Conditional indirect effect: *Self‐efficacy x total received social support*	−.132*	.058	.023
	**Index of moderated mediation**	**Boot *SE***	**Boot 95% CI**
*Total received social support*	.039	.018	(.007, .078)
**Conditional indirect effect** **At different values of moderator** **(total received social support)**	**Boot indirect effect**	**Boot *SE***	**Boot 95% CI**
1.0	−.230	.071	(−.378, −.101)
2.0	−.191	.056	(−.308, −.088)
3.0	−.152	.043	(−.239, −.072)
4.0	−.112	.034	(−.182, −.051)
5.0	−.073	.035	(−.147, −.011)
5.109^a^	−.069	.035	(−.144, −.006)
6.0	−.034	.043	(−.123, .050)

*Note*: *n* = 536 (Hypothesis 3)/504 (Hypothesis 4); Bootstrap sample size = 10,000.

^a^ Moderator value defining the Johnson‐Neyman significance region (79.37% below and 20.63% above).

* *p* < .05.

Both interaction terms between instrumental support and support network size were not significant (conditional effect on toilet training intensity: *B* = .004, *p* = .867; conditional effect on self‐efficacy: *B* = .020, *p* = .596). This indicates that support network size does not moderate the direct effect of instrumental support on toilet training nor does it moderate the effect of instrumental support on caregiver's perceived self‐efficacy. Moreover, the index of moderated mediation did not support the claim that support network size moderates the indirect effect of instrumental support on toilet training through self‐efficacy (*B* = .001, Boot 95% CI: −.017, .020). As there was no evidence of moderation, we did not probe either of the two interactions.

### Hypothesis 4

Lastly, we wanted to determine if social support buffers caregivers against stress when it comes to toilet training. The average perceived stress score among caregivers was 2.92 (SD: 0.52) out of 5, indicating moderate to high levels of stress. We first examined whether or not a caregiver's perceived level of stress is negatively associated with her intensity of toilet training, be it directly or through diminishing her sense of self‐efficacy. The simple mediation analysis revealed no direct effect of perceived stress on toilet training intensity. However, perceived stress did have a negative indirect effect on toilet training intensity through self‐efficacy (*B =* −.137, Boot 95% CI: −.215, −.070).

Next, we examined if this deleterious effect of stress on toilet training is indeed “buffered,” or *moderated*, by the caregiver's received social support. We hypothesized that the negative indirect effect of perceived stress on toilet training intensity would be weaker for caregivers who have a high level of received social support compared to caregivers with low levels of support. The interaction term between received social support and self‐efficacy was significant (*B* = −.132, *p* < .05), indicating the effect of self‐efficacy on toilet training intensity depends upon the caregiver's received social support. The index of moderated mediation was also significant, confirming that a caregiver's level of received social support moderates the indirect effect of perceived stress on toilet training through self‐efficacy (*B* = .039, bootstrapped 95% CI: .007, .078). We then probed the interaction and identified a significance region between the received social support values of 1.0 to 5.109. As predicted, the negative association between perceived stress and toilet training intensity weakens as levels of received social support increase. The statistical diagram and corresponding unstandardized regression coefficients of the hypothesized conditional effect are presented in Figure 1d and the interaction term is presented in Table 3.

## DISCUSSION

To our knowledge, this is the first study to examine the role of social support in child toilet training for rural Indian caregivers. We used mediation and conditional process analyses to test how this social resource could influence caregivers' toilet training efforts. We found specific types of social support aid toilet training through three mechanisms: directly, by improving self‐efficacy, and by helping to buffer against life stress. Both informational and instrumental support had a direct effect on toilet training intensity while emotional support had no effect. Instrumental support also aided toilet training indirectly through improving a caregiver's perception of her ability to take on toilet training with her child. Additionally, these effects of instrumental support were not dependent upon how many sources of support the caregiver had in her life. Lastly, we found perceived stress negatively influences a caregiver's toilet training efforts through diminishing her sense of self‐efficacy, but social support buffers against this deleterious effect. These findings offer useful insights on support programs for rural Indian caregivers and also help expand the evidence‐base on how social support functions to another childcare practice.

Other studies on parenting and childcare practices have demonstrated the stress‐support matching hypothesis. A study among Korean mothers living in the United States showed instrumental support, but not emotional support, led to a less authoritarian parenting style through improving maternal psychological well‐being (Seo et al., 2017). Another study among Mexican American parents found Mexico‐born parents are less depressed than their US‐born counterparts leading to better asthma control for their children, but this protective effect was specifically dependent upon high levels of instrumental support, with no moderating effect by informational or emotional support (Weinstein et al., 2020). While there are very few studies on the types of social support parents desire with toilet training, this study adds to emerging evidence that instrumental support may prove critical. In the one qualitative study by Van Aggelpoel et al. (2019), Belgian parents were asked about the kinds of informational support they seek with toilet training. But parents also described the importance of receiving tangible, instrumental help from others, including grandparents and teachers. As one parent explained, “Everyone has to join in” (Van Aggelpoel et al., 2019). This growing evidence‐base for the stress‐support matching hypothesis highlights the importance of understanding which types of social support caregivers desire for a given childcare practice in order to design effective programming. Moreover, more research is needed to better understand the significance of instrumental support in toilet training and the support needs of different caregivers involved in the training process, such as comparing the needs of mothers, fathers and grandmothers.

We originally hypothesized a larger support network may allow for more opportunities to receive support but relationship characteristics and satisfaction of the support given may be more important factors. Relationship characteristics likely dictate the type of support available where stronger relationships, such as those with partners, family and friends, are able to provide more intensive and resource‐heavy forms of support (Wellman & Wortley, 1990). For example, a qualitative study with Polish migrant mothers living in Dublin uncovered that the mothers heavily relied upon family members, particularly grandparents, to provide instrumental childcare support, despite geographical distance (Bojarczuk & Mühlau, 2018). The mothers tended to only seek support from local social ties in times of crisis (Bojarczuk & Mühlau, 2018). It is possible that we found no moderating effect of support network size on toilet training because, no matter the size, the Indian caregivers' networks shared similar characteristics—mostly comprised of family members, especially the husband and mother‐in‐law. It is also possible a single family member could adequately meet a caregiver's support needs. Instead of network size, future research should examine the moderating effect of the strength of the relationship between caregivers and their primary sources of support. In the Indian context, the relationship between a woman and her mother‐in‐law, or the grandmother of the child, may prove critical for childcare support (Seymour, 1999). These findings also demonstrate the need for social support programs to consider not only the type of support desired by caregivers but also the source best suited to give that support, whether it is a relationship already in the caregiver's natural network that the program can work to strengthen or a new social tie the program can introduce (Gottlieb, 2000).

Since the 1980s there has been a call for empirical studies to test the theorized mechanisms for how social support functions. In particular, for studies to examine the behavioral mechanism of social support—its boosting effect on self‐efficacy which is a precursor for behavioral action—and the cognitive mechanism of social support—its strengthening of coping and appraisal reasoning to buffer against stress. Here we provide evidence for both functions of social support when it comes to toilet training, but we also uncovered other potential behavioral functions. While we showed self‐efficacy mediates the effect of instrumental support on toilet training, we also found instrumental and informational support have a *direct* effect. These forms of support led caregivers to more intensely take on toilet training through other avenues beyond building confidence alone. It could be the provision of instrumental support, such as another family member helping with cooking or cleaning tasks, simply creates more opportunity for the caregiver to practice toilet training with her child. This might be particularly relevant for rural Indian mothers who likely face difficult time‐constraints between childcare tasks and their domestic and agricultural workload. Similarly, informational support like advice or feedback may simply foster better action knowledge for the caregiver on how to do toilet training. Future research should explore these other potential mechanisms for how social support leads to better behavioral performance when it comes to demanding childcare tasks.

This study has several limitations. First, this is a cross‐sectional study design which limits our ability to test the actual directionality of the examined social support mechanisms (as shown in Figure 1). Second, we measured *received* social support, asking caregivers about support they had experienced in the last week, rather than *perceived* social support. Methodologists suggest perceived social support is a more stable construct that is a better predictor of self‐efficacy since a person's confidence can be improved by simply perceiving support is available to them (Cohen et al., 2000). This may have limited our ability to detect a stronger mediating effect for self‐efficacy. Third, the data are not all temporally matched since the PSS measured caregiver's perceived stress during the last *month* rather than the last *week*, as was done in the other metrics. Finally, we constructed our own metrics for received social support and perceptions of self‐efficacy in toilet training. We took a rigorous approach, following many of the promoted steps for scale development (Boateng et al., 2018), but more research is needed to further test the reliability and validity of these metrics.

### Conclusion

Over the last several decades India has seen a dramatic increase in access to sanitation, especially in rural areas (JMP, 2019). As more families adopt toilet use, child toilet training is becoming a necessity. This is the first study to examine the role of social support in toilet training for rural Indian caregivers and offers practical implications for future programming. Our results suggest programs for Indian caregivers should focus on providing instrumental and informational support on toilet training. We also found the size of a caregiver's support network does not influence toilet training. Programs might instead consider the sources best suited to meet the caregiver's different support needs: this could include strengthening an existing relationship, potentially with husbands or mothers‐in‐law who can offer instrumental support, and/or introducing a new social tie like a community health worker who can offer toilet training advice. Lastly, we found life stress had a negative effect on caregivers' toilet training efforts through diminishing self‐efficacy, but this effect was buffered by social support. As the sanitation sector in India switches focus to child toilet use, programs should consider social support techniques to ensure Indian mothers and caregivers are not overburdened by toilet training but instead feel confident and are successful.

## ETHICS STATEMENT

This study received ethics approval from the Institutional Review Board (IRB) at Emory University (IRB00115339) in USA and the Independent Ethics Committee at Xavier University Bhubaneswar (220519) in Odisha, India. All participants were 18 years or older and provided their informed, verbal consent before being engaged in the study.

## CONFLICT OF INTEREST

The authors declare no conflicts of interest.

## Supporting information


**Data S1** Supporting Information

## Data Availability

The data used in this study are available in the Dataverse open data repository (https://doi.org/10.15139/S3/EKDFDL).
