# Supplementary material for: Caregiver social support and child toilet training in rural Odisha, India: What types of support facilitate training and how?
Source: Appl Psychol Health Well Being. 2021 Oct 19;14(2):413–33. doi: 10.1111/aphw.12311 (PMC9297906; doi:10.1111/aphw.12311)
Supplement: Supplementary file 1 — Data S1 Supporting Information [file APHW-14-413-s001.docx]

**SUPPLEMENTAL TABLES AND FIGURES**

**Table S1. Survey items used in the analysis**

| **Measure** | **Survey Item** | **Response Options** |
| --- | --- | --- |
| **Toilet training intensity** | During the last week, when (name of child) needed to defecate, how often did you take (name of child) to the latrine and teach them how to use it? | 1 = (almost) never (0%) 2 = seldom (25%) 3 = sometimes (50%) 4 = often (75%) 5 = (almost) always (100%)  98 = Refused  99 Participant could not understand |
| **Self-efficacy** | How confident are you in your ability to successfully teach your child how to use a latrine? | 1 = not confident 2 = a little confident 3 = confident 4 = quite confident 5 = very confident 98 = Refused  99 = Participant could not understand |
|  | If your child needs to defecate in the nighttime and needs your help, how confident are you that you would go and help your child use the latrine? |  |
|  | How confident are you in your ability to teach your child how to use the latrine when there is a water shortage? |  |
|  | How confident are you in your ability to continue teaching your child how to use a latrine when your child refuses to use the latrine (for example child cries or won't enter the latrine)? |  |
| **Prompt - Received Social Support Sub-Survey:** *For the next set of questions, think about the different people in your life such as family members, neighbors and friends. What help did you receive in the last week with teaching your child to defecate in the latrine?* | | |
| **Received Emotional Support** | In the last week, someone **listened to me** when I needed to talk about my struggles with teaching my child to defecate in the latrine. | 1 = strongly disagree 2 = disagree 3 = somewhat disagree 4 = somewhat agree 5 = agree 6 = strongly agree 98 Refused  99 Participant could not understand |
|  | In the last week, someone **comforted me** when I was struggling with teaching my child to defecate in the latrine. |  |
|  | In the last week, someone **expressed to me they understood** what it is like to struggle with teaching your child to defecate in the latrine. |  |
|  | In the last week, someone **encouraged me** to teach my child to defecate in the latrine. |  |
|  | In the last week, someone told me I could **rely on them** for help with teaching my child to defecate in the latrine. |  |
|  | In the last week, someone expressed to me that they think I am a **good mother** (father / caregiver). |  |
| **Received Instrumental Support** | In the last week, someone **reminded/encouraged my child** to defecate in the latrine. |  |
|  | In the last week, someone **helped my child defecate in the latrine** when I was not available to do it. |  |
|  | In the last week, someone **helped with the cooking or cleaning** for me so I could go help my child defecate in the latrine. |  |
|  | In the last week, someone **collected water** for me that I needed to help my child defecate in the latrine. |  |
| **Received Informational Support** | In the last week, someone **gave me advice** on how to teach my child to defecated in the latrine. |  |
|  | In the last week, someone helped me **make a decision** about how to teach my child to defecate in the latrine. |  |
|  | In the last week, someone **gave me feedback** on how I was teaching my child to defecate in the latrine. |  |
| **Support network size** | For the statements that you agreed to, who were the people that provided you this support? | 1 = Husband / wife  2 = Mother-in-law  3 = Father-in-law  4 = Mother  5 = Father  6 = Aunt  7 = Uncle  8 = Daughter-in-law  9 = Son-in-law  10 = Sister-in-law  11 = Brother-in-law  12 = Sister  13 = Brother  14 = Daughter  15 = Son  16 = Granddaughter  17 = Grandson  18 = Anganwadi*  19 = ASHA+  20 = Neighbor  21 = Village elder  88 = Other  77 = Not applicable - participant did not agree to any of the statements  98 = Refused |
| **Prompt - Perceived Stress Scale Prompt:** *I would like to ask you some questions about your feelings and thoughts during the last month. In each case, you will be asked to indicate how often you felt or thought a certain way. Although some of the questions are similar, there are differences between them and you should treat each one as a separate question. The best approach is to answer each question fairly quickly.* | | |
| **Perceived Stress (Cohen’s PSS-10)** | In the last month, how often have you been upset because of something that happened unexpectedly? | 1 = never 2 = almost never 3 = sometimes 4 = fairly often 5 = very often 98 = Refused  99 = Participant could not understand |
|  | In the last month, how often have you felt that you were unable to control the important things in your life? |  |
|  | In the last month, how often have you felt nervous and "stressed"? |  |
|  | In the last month, how often have you felt confident about your ability to handle your personal problems |  |
|  | In the last month, how often have you felt that things were going your way? |  |
|  | In the last month, how often have you found that you could not cope with all the things that you had to do? |  |
|  | In the last month, how often have you been able to control irritations in your life? |  |
|  | In the last month, how often have you felt that you were on top of things? |  |
|  | In the last month, how often have you been angered because of things that happened that were outside of your control? |  |
|  | In the last month, how often have you felt difficulties were piling up so high that you could not overcome them? |  |

*Anganwadi worker runs the local government-run childcare center, known as an “Anganwadi”

+ASHA (Accredited Social Health Worker) is a community health worker under the National Rural Health Mission instituted by the Government of India in 2005

**Table S2.** Participant and household demographics

| ***Participant characteristics*** | **n** | **%*** |
| --- | --- | --- |
| **Primary caregiver of the child <5** | 502 | 88.1% |
| **Relation to child <5** |  |  |
| Mother | 494 | 86.7% |
| Father | 44 | 7.7% |
| Grandmother | 26 | 4.6% |
| Grandfather | 1 | 0.2% |
| Aunt | 5 | 0.9% |
| **Age** |  |  |
| 18 to 30 years old | 421 | 73.9% |
| 31 to 40 years old | 114 | 20.0% |
| 41 to 50 years old | 20 | 3.5% |
| >=51 years old | 15 | 2.6% |
| **Currently married** | 543 | 97.3% |
| Mean age at time of marriage [Mean (SD)] | 19.2 | 3.0 |
| **Education** |  |  |
| Never attended school | 129 | 23.1% |
| Anganwadi (preschool) | 0 | 0.0% |
| Primary (grades 1 to 5) | 93 | 16.7% |
| Upper primary (grades 6-8) | 101 | 18.1% |
| Secondary (grades 9-10) | 165 | 29.6% |
| Senior secondary (grades 11-12) | 42 | 7.5% |
| Graduate/post-graduate (13+) | 26 | 4.7% |
| Refused/don't know | 2 | 0.4% |
| **Occupation** |  |  |
| Unemployed (No work that earns money) | 278 | 49.8% |
| Self-Employed  (Work in home: agriculture, potter, forest) | 218 | 39.1% |
| Employed  (Work outside of home: laborer, factory) | 62 | 11.1% |
| Student | 0 | 0.0% |
| **Defecated in toilet last time** | 434 | 77.9% |
| ***Household characteristics*** | **n** | **%** |
| **Number of children (ages 0 to <5 years)**** |  |  |
| One child | 467 | 81.9% |
| Two children | 98 | 17.2% |
| Three children | 5 | 0.9% |
| **Mean household size** [Mean (SD)] | 5.8 | 2.2 |
| **Religion** |  |  |
| Hindu | 443 | 79.4% |
| Christian | 112 | 20.1% |
| No religion | 1 | 0.2% |
| Refused/don't know | 2 | 0.4% |
| **Caste** |  |  |
| Scheduled tribe | 144 | 25.8% |
| Other backward caste (OBC) | 193 | 34.6% |
| Scheduled caste | 45 | 8.1% |
| General | 99 | 17.7% |
| Other | 39 | 7.0% |
| Don't know/Refused | 38 | 6.8% |
| **Have BPL, Antyodaya and/or ration card+** | 482 | 84.6% |
| **Household water and sanitation facilities** |  |  |
| Pour-flush toilet | 560 | 100% |
| Piped water (functional) | 495 | 88.4% |
| Bathing room | 544 | 97.1% |
| **Share toilet with other households** | 25 | 4.5% |

*The denominator is 570 for most demographic characteristics but not all. This resulted from some respondents ending the survey early and not answering demographic questions that appeared later in the survey. However, all respondents answered the toilet training intensity question and thus were included in the analysis. The following demographic characteristics had a denominator less than 570: Respondent toilet use (n = 557) / Marriage status, education, occupation, household size, religion, and caste (n = 558) / Household water and sanitation facilities, sharing toilet (n = 560)

**There were 19 caregivers who had more than one child currently being taught to use the toilet. Among these, only 8 caregivers reported a different level of toilet training intensity for each child - the toilet training intensity for the youngest child was included in the analysis.

+Ration cards are a government issued document that entitle poor households to food subsidies under the National Food Security Act (NFSA) 2013. Prior to the NFSA, there was a different food assistance program that included a ‘Below Poverty Line’ (BPL) ration card and ‘Antyodaya Anna Yojana’ ration card. As a result, households use these various names— “BPL”, “Antyodaya,” and “ration card”—to indicate if they have a ration card and thus qualify for food assistance.


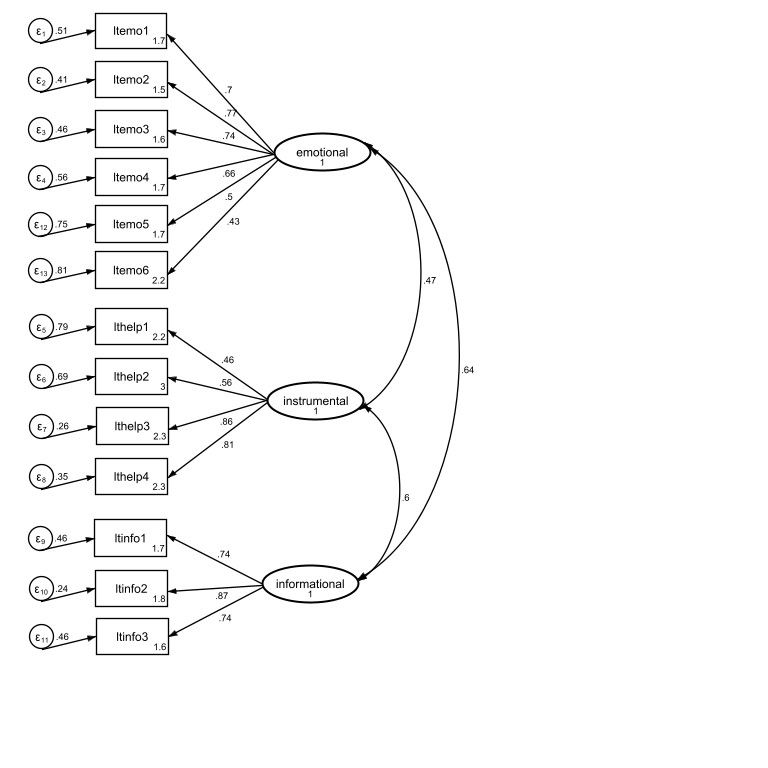


**Figure S1. Hypothesized social support 3-factor model**

**Table S3. Social support CFA results for both original 13-item model and refined 10-item model**

| **Factors** | **Associated items** | **Original CFA -**  **Standardized factor loadings*** | **Refined CFA -**  **Standardized factor loadings*** |
| --- | --- | --- | --- |
| Factor 1:  Emotional support | In the last week, someone listened to me when I needed to talk about my struggles with teaching my child to defecate in the latrine. | .702 | .721 |
|  | In the last week, someone comforted me when I was struggling with teaching my child to defecate in the latrine. | .766 | .803 |
|  | In the last week, someone expressed to me they understood what it is like to struggle with teaching your child to defecate in the latrine. | .737 | .734 |
|  | In the last week, someone encouraged me to teach my child to defecate in the latrine. | .664 | .634 |
|  | In the last week, someone told me I could rely on them for help with teaching my child to defecate in the latrine. | .498 | - |
|  | In the last week, someone expressed to me that they think I am a good mother (father / caregiver). | .433 | - |
| Factor 2:  Instrumental support | In the last week, someone reminded/encouraged my child to defecate in the latrine. | .457 | - |
|  | In the last week, someone helped my child defecate in the latrine when I was not available to do it. | .557 | .541 |
|  | In the last week, someone helped with the cooking or cleaning for me so I could go help my child defecate in the latrine. | .857 | .892 |
|  | In the last week, someone collected water for me that I needed to help my child defecate in the latrine. | .807 | .802 |
| Factor 3:  Informational support | In the last week, someone gave me advice on how to teach my child to defecated in the latrine. | .737 | .735 |
|  | In the last week, someone helped me make a decision about how to teach my child to defecate in the latrine. | .870 | .872 |
|  | In the last week, someone gave me feedback on how I was teaching my child to defecate in the latrine. | .737 | .737 |

n = 565; estimation method = MLMV; *All unstandardized factor loadings were significant at *p* <.001


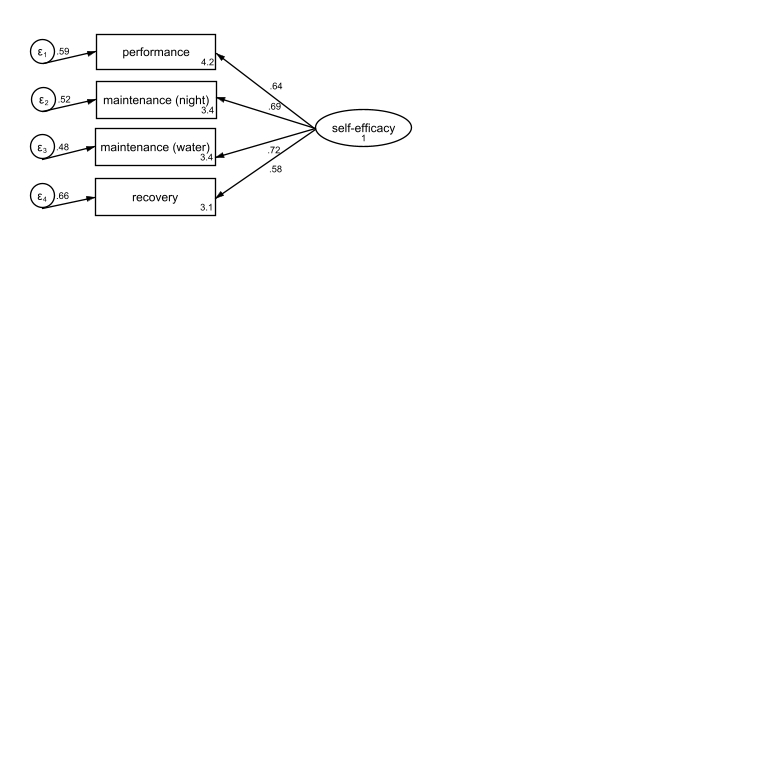


**Figure S2. Hypothesized self-efficacy 1-factor model**

**Table S4. Self-efficacy CFA results**

| **Factors** | **Associated items** | **Standardized factor loadings*** |
| --- | --- | --- |
| Factor 1:  Self-efficacy | *Behavioral performance self-efficacy:* How confident are you in your ability to successfully teach your child how to use a latrine? | .644 |
|  | *Maintenance self-efficacy:* If your child needs to defecate in the nighttime and needs your help, how confident are you that you would go and help your child use the latrine? | .691 |
|  | *Maintenance self-efficacy:* How confident are you in your ability to teach your child how to use the latrine when there is a water shortage? | .721 |
|  | *Recovery self-efficacy:* How confident are you in your ability to continue teaching your child how to use a latrine when your child refuses to use the latrine (for example child cries or won't enter the latrine)? | .583 |

n = 575; estimation method = MLMV; *All unstandardized factor loadings were significant at *p* <.001
